# The Role of Bronchoscopy in the Diagnosis of Interstitial Lung Disease: A State-of-the-Art Review

**DOI:** 10.3390/jcm14093255

**Published:** 2025-05-07

**Authors:** A. Rolando Peralta, Al Muthanna Shadid

**Affiliations:** 1 Interventional Pulmonology, Division of Pulmonary and Critical Care, Henry Ford Hospital, Detroit, MI 48202, USA; 2 Division of Pulmonary and Critical Care, Henry Ford Hospital, Detroit, MI 48202, USA; ashadid1@hfhs.org

**Keywords:** bronchoscopy, interstitial lung disease, transbronchial lung biopsy, transbronchial lung cryobiopsy

## Abstract

The diagnostic evaluation of interstitial lung diseases (ILDs) remains challenging due to their heterogeneous etiologies and overlapping clinical and radiographic patterns. A confident diagnosis often necessitates histopathological sampling, particularly when high-resolution computed tomography and serologic assessments are inconclusive. While surgical lung biopsy (SLB) has long been considered the diagnostic gold standard, its invasiveness, associated morbidity, and limited feasibility in high-risk patients have driven the pursuit of less invasive alternatives. Here, we review the current applications, diagnostic yield, procedural techniques, and complications of several bronchoscopic modalities. Bronchoalveolar lavage (BAL) aids in characterizing inflammatory profiles and differentiating among conditions such as hypersensitivity pneumonitis, sarcoidosis, and eosinophilic pneumonia. Endobronchial biopsies (EBBs) and endobronchial ultrasound transbronchial needle aspiration (EBUS-TBNA) are valuable in diagnosing granulomatous diseases with lymphadenopathy. Transbronchial lung biopsy (TBLB) is effective for peribronchial and centrilobular diseases but is limited by small sample size and tissue distortion. Transbronchial lung cryobiopsy (TBC) enables acquisition of larger, well-preserved parenchymal tissue samples from the peripheral lung. Over recent years, studies have demonstrated that TBC, when interpreted within a multidisciplinary discussion (MDD), achieves diagnostic concordance rates with SLB exceeding 75%, and up to 95% in cases where high diagnostic confidence is reached. When performed in experienced centers using standardized protocols, TBC is considered a viable first-line histopathologic tool in the diagnostic evaluation of ILD. Adequate training and standardization of the TBC procedure are needed to ensure low complication rates and a high yield.

## 1. Introduction

**Figure 1:**
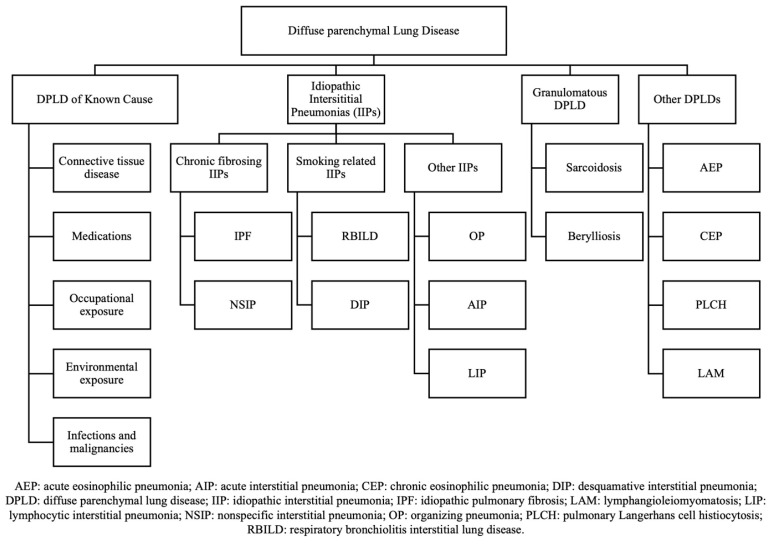
General classifications of diffuse parenchymal lung diseases.

Interstitial lung diseases (ILDs), sometimes referred to as diffuse parenchymal lung diseases (DPLDs), encompass a heterogeneous group of disorders characterized by inflammation and fibrosis which may affect the pulmonary parenchyma, alveolar epithelium, capillary endothelium, and the interstitial space. DPLDs are categorized into those with an identifiable cause, such as occupational and environmental exposures, adverse drug reactions, and related to connective tissue diseases; those without an identifiable cause, also known as idiopathic interstitial pneumonias (IIPs); granulomatous DPLDs like sarcoidosis; and other forms of DPLP [Figure 1] [1]. Among the IIPs, idiopathic pulmonary fibrosis (IPF) is the most prevalent and severe subtype. IPF is characterized by a usual interstitial pneumonia (UIP) pattern on imaging and histopathology and typically affects older adults, with a prognosis that remains poor despite advances in therapeutic options [2,3].

Reaching a confident diagnosis of DPLD requires a comprehensive assessment of the patient’s history, physical exam findings, pulmonary function tests, serologic testing, and chest imaging. A multidisciplinary discussion (MDD) involving pulmonologists, radiologists, and pathologists is critical in achieving accurate diagnoses [4,5,6]. This approach is particularly important when a confident diagnosis cannot be made based on clinical and imaging data alone.

Depending on the confidence in the diagnosis, more invasive procedures are required to obtain further objective data. Surgical lung biopsy (SLB) remains the gold standard in the diagnosis of unspecified ILD and provides a specific diagnosis in most cases. However, SLB is invasive, requires general anesthesia, and carries a significant risk of complications. Less invasive bronchoscopic techniques have varying additive diagnostic roles in some cases. Bronchoscopy is often preferred when less invasive techniques with fewer complications can provide useful diagnostic information, especially when integrated within a multidisciplinary framework. Bronchoalveolar lavage (BAL) helps rule out infection and can provide additional findings suggestive of specific diseases. Transbronchial lung biopsies (TBLBs) are particularly beneficial in diagnosing centrilobular disease processes [7]. Endobronchial lung biopsy (EBB) has a limited role, mainly in diagnosing sarcoidosis when mucosal abnormalities are noted [8]. Transbronchial lung cryobiopsy (TBC) is a relatively newer technique that yields larger-sized samples with fewer crush artifacts compared to TBLB [9]. In the last decade, TBC has emerged as a potential alternative to SLB particularly when combined with MDD [6,10].

This review aims to explore the current role of different bronchoscopic techniques in the diagnosis of ILD. A special focus is given to TBC due to the recent expansion in the literature.

### 1.1. The Role of Bronchoalveolar Lavage (BAL)

BAL is a minimally invasive diagnostic procedure that involves the instillation of sterile saline into a targeted lung segment and its subsequent retrieval for analysis. BAL allows for the examination of cellular and non-cellular components within the alveolar space. While BAL alone is not diagnostic, it can aid in the diagnosis when combined with clinical and radiographic data. However, in certain conditions, BAL findings may be highly diagnostic or even pathognomonic.

In healthy individuals, BAL fluid typically consists of 72–96% macrophages, 2–26% lymphocytes, 0–4% neutrophils, and 0–1% eosinophils. These reference values remain consistent across different ages, genders, seasons, and lung collection sites [11,12]. A BAL cell differential with >15% lymphocytes, >3% neutrophils, >1% eosinophils, or >0.5% mastocytes indicates a predominant inflammatory pattern. Epithelial cells of >5% suggest a suboptimal sample and interpretation should be performed with caution [13].

An important role of BAL in ILD includes identifying and ruling out conditions such as infections or malignancies, which can mimic the clinical and radiological presentations of ILDs [12,14].

Several guidelines have highlighted the role of cellular patterns from BAL. A lymphocytic predominant pattern can be suggestive of hypersensitivity pneumonitis (HP) over IPF in newly diagnosed ILD [15,16]. Sarcoidosis also produces a lymphocytic pattern with a markedly elevated CD4/CD8 ratio within the lymphocyte population. HP typically has higher lymphocytic predominance than sarcoidosis along with a normal CD4/CD8 ratio [17].

Other less common diseases that produce lymphocyte-predominant fluid analysis include lymphocytic interstitial pneumonia (LIP), connective tissue disease-associated ILDs, drug-induced pneumonitis, and chronic beryllium disease (CBD) which is differentiated from sarcoidosis using the beryllium lymphocyte proliferation test [18,19,20,21].

Neutrophilic patterns can be seen in bacterial and aspiration pneumonia [22]. Acute respiratory distress syndrome (ARDS) and diffuse alveolar damage (DAD) have neutrophilic patterns due to significant infiltration resulting in alveolar injury. Neutrophilic patterns can be also seen in acute exacerbations of IPF or in advanced stages of IPF, which suggest poor prognosis [23,24].

Conditions that can present with eosinophilic predominance in BAL fluid include acute eosinophilic pneumonia (AEP), chronic eosinophilic pneumonia (CEP), allergic bronchopulmonary aspergillosis (ABPA), eosinophilic granulomatosis with polyangiitis (EGPA), formerly known as Churg–Strauss syndrome, in addition to parasitic infections, such as ascariasis and strongyloidiasis [25,26,27,28,29]. Table 1 summarizes general patterns of cellular analysis of BAL.

In certain conditions, BAL findings can be highly diagnostic or even pathognomonic. Pulmonary alveolar proteinosis (PAP) presents with characteristically milky, turbid fluid containing PAS-positive acellular globules and abnormal foamy macrophages [30]. Pulmonary Langerhans cell histiocytosis (PLCH) is suggested by a CD1a+ Langerhans cell count exceeding 4% of total BAL cells, a highly specific finding, though sensitivity is around 50% [31]. Diffuse alveolar hemorrhage (DAH) is indicated by a progressively bloody return on serial aliquots, with fluid analysis revealing hemosiderin-laden macrophages and free red blood cells. A thorough history and additional workup can aid in evaluating the underlying cause of the DAH [32] [Table 2].

BAL is a relatively cost-effective and widely available technique that can be used in low-resource due to minimal equipment requirements and straightforward procedural nature [33].

### 1.2. The Role of Endobronchial Biopsy (EBB)

EBBs are obtained during flexible bronchoscopy and involve the collection of tissue samples from the bronchial mucosa using forceps. EBB does not provide sufficient lung parenchymal tissue, so its role in ILD is limited to granulomatous diseases, mainly for sarcoidosis and CBD. The latter is confirmed by the beryllium lymphocyte proliferation test [18].

The reported yield of TBLB in suspected sarcoidosis is 61.8%. The addition of EBB increases the overall diagnostic yield of fiberoptic bronchoscopy by 20.6% [34]. When endobronchial ultrasound-guided transbronchial needle aspiration (EBUS-TBNA) is combined with TBLB and EBB, the diagnostic yield reaches 89.7% [12,35]. Like BAL, EBB is feasible and widely available in low-resource settings [33].

### 1.3. The Role of Transbronchial Lung Biopsy (TBLB)

TBLB is another minimally invasive procedure performed during bronchoscopy. Using a flexible fiberoptic bronchoscope, forceps retrieve small samples of lung parenchyma, commonly under fluoroscopic guidance to ensure accurate sampling and minimize complications.

TBLB has a limited role in the diagnosis of fibrotic ILD. Although specimens may be considered adequate in most cases, the overall utility is limited by the size of the specimen and the presence of crush artifacts, and a final accurate diagnosis is only reached in 20–30% when the TBLB is combined with clinical and radiologic data [36]. Conversely, it may be useful in diagnosing ILDs with centrilobular involvement, where small samples of lung tissue are sufficient to make the diagnosis [7,12]. It is particularly effective in diagnosing sarcoidosis and CBD by identifying non-caseating granulomas [8]. TBLB can also aid in diagnosing HP by detecting characteristic histopathological features such as granulomas or giant cells, with a diagnostic yield of 37% [16,37]. In organizing pneumonia (OP), TBLB helps identify intraluminal plugs of loose connective tissue within alveolar spaces, ducts, and sometimes bronchioles [38]. A study by Poletti et al. demonstrated that TBLB had a sensitivity of 64% and a specificity of 86% for diagnosing organizing pneumonia, with a positive predictive value of 94% and a negative predictive value of 40% [39]. This differentiation is crucial, as eosinophilic pneumonia is histopathologically distinguished by eosinophilic infiltrates [40].

The diagnostic yield of TBLB varies in cystic lung diseases. In PLCH, it is relatively low (10–40%) due to the patchy lesion distribution and the small biopsy sample size [41]. In contrast, TBLB has a higher diagnostic yield in lymphangioleiomyomatosis (LAM), with reported yields ranging from 57% to 78.9%, achieving better success in cases of greater cystic lung destruction and extensive sampling [42,43]. TBLB offers a less invasive approach to SLB and is recommended when the typical clinical features (tuberous sclerosis complex, angiomyolipomas, chylous effusions, lymphangioleiomyomas, serum vascular endothelial growth factor-D (VEGF-D) ≥ 800 pg/mL) are absent and a histopathological confirmation is required [42].

TBLB can also assist in diagnosing PAP; however, the combined use of BAL analysis and serum GM-CSF autoantibody measurement can often eliminate the need for histologic confirmation [44].

Despite its poor sensitivity and limited utility when compared to TBC and SLB. TBLB remains relevant due to its lower complication risks, especially regarding bleeding [45]. TBLB is also more accessible and less resource-intensive, requiring only standard bronchoscopic tools and no advanced training [46]. TBLB is feasible but requires fluoroscopic guidance, which may limit its availability in some low-resource settings [33].

### 1.4. The Role of Endobronchial Ultrasound-Guided Transbronchial Needle Aspiration (EBUS-TBNA)

EBUS-TBNA is a minimally invasive procedure used to obtain tissue samples from the mediastinal and hilar lymph nodes as well as parabronchial masses. An ultrasound transducer at the tip of a bronchoscope allows for real-time visualization and guidance for needle aspiration.

The American College of Chest Physicians (CHEST) recommends EBUS-TBNA for the diagnosis of conditions such as sarcoidosis and tuberculosis when there is mediastinal and/or hilar adenopathy, as well as for the initial evaluation in suspected lymphoma cases [47]. The procedure has transformed the diagnostic approach to mediastinal and hilar diseases, particularly in non-small cell lung cancer, due to its high sensitivity and negative predictive value [48].

EBUS-TBNA is useful in the diagnosis of sarcoidosis, where it has a high sensitivity and specificity [49,50]. Compared to conventional transbronchial needle aspiration (cTBNA), EBUS-TBNA has a significantly higher diagnostic yield (83.3% vs. 53.8%) [51].

The 2020 American Thoracic Society (ATS) guidelines for the diagnosis of sarcoidosis recommend EBUS-TBNA as the initial procedure for lymph node sampling in patients with suspected sarcoidosis with mediastinal and/or hilar lymphadenopathy, emphasizing its advantages over more invasive procedures like mediastinoscopy. It has a diagnostic yield of 87% and is associated with fewer complications and lower costs compared to mediastinoscopy [8]. Furthermore, combining EBUS-TBNA with TBLB and EBB has been shown to further increase its diagnostic yield [35]. Limitations of EBUS-TBNA include inadequate tissue sampling and false negatives which can be influenced by technical challenges, such as patient movement, cough, insufficient sedation, or anatomical factors including the lymph node size and location [52,53].

### 1.5. Transbronchial Lung Cryobiopsy (TBC)

Cryotherapy use in pulmonary medicine dates back to the 1980s. The procedure was initially intended to treat benign and malignant airway obstruction via a rigid or flexible cryoprobe [54,55,56]. Babiak reported the first use of a flexible cryoprobe through a flexible bronchoscope with the objective of obtaining TBC in 2009 [57]. Since its first use in pulmonary medicine, extensive research has positioned TBC as an alternative diagnostic and therapeutic tool to be used during bronchoscopy.

SLB remains the gold standard in the diagnosis of unspecified ILD and provides a specific diagnosis in 88% of cases. It is generally recommended in patients who tolerate single-lung ventilation and whose physiologic limitations and comorbidities do not render the procedure unsafe. Respiratory infection, exacerbations, and persistent air leaks occur in 5–6% of cases. Delayed wound healing, bleeding, and neuropathic pain occur less frequently. The overall mortality is 3.5% with a procedural mortality of 1.7% [58]. TBC is now recommended as an alternative to SLB in patients with unspecified ILD when performed in centers of excellence that have instituted protocols to minimize the risks and maximize the potential diagnostic value of the procedure. This is based on the comparable histopathological yield and more favorable safety profile [5,59].

### 1.6. TBC—Yield in ILD

The diagnosis of ILD is a multidisciplinary effort, requiring a detailed medical history, HRCT, and serologic testing. The use of an MDD for the detailed review of the available information is a core step in the workup of patients with ILD. A tissue biopsy is considered in those cases in which the diagnosis remains unspecified [58]. At any point during the evaluation, a level of confidence can be assigned based on the perceived likelihood of a given clinical diagnosis. In this ontological classification, a diagnosis can be considered confident, provisional high-confidence, provisional low-confidence, or unclassifiable (≥90%, 70–89% 51–69%, and ≤50% confidence, respectively) [60].

When discussing the utility of tissue sampling for ILD, we need to differentiate between histopathological yield and clinical diagnostic yield. The former refers to the number of procedures that yielded a histopathological diagnosis divided by the total number of procedures, and the latter refers to a more comprehensive assessment of the histology and pertinent clinical and radiologic findings at the MDD level. Although the yield of TBC is higher than TBLB for most ILD diagnoses [45,61], studies addressing the overall yield compared to SLB show variable results [62,63]. The histological yield of TBC for ILD in a recent systematic review was 80% (95% CI, 76–84%). This increased to 85% when three or more samples were collected, compared to 77% with fewer samples [64]. The COLDICE study showed a raw histopathological agreement of 70.8% for TBC and SLB, and a 76.9% clinical diagnosis agreement after MDD. For cases where a “definite” or “provisional high confidence” diagnosis was reached after TBC + MDD, the concordance between TBC and SLB was as high as 95%. In cases where only “provisional low confidence” was reached after TBC + MDD, SLB reclassified 23% of those [10].

When obtaining lung tissue is deemed necessary, a step-up approach consisting of TBC first followed by SLB in non-diagnostic cases may be used [59]. In a recent RCT, this approach showed a diagnostic yield of 82% for TBC + MDD alone, which increased to 89% when SLB was subsequently performed in the inconclusive cases. This is compared to an 88% diagnostic yield for immediate SLB + MDD. Serious adverse events occurred less frequently in the step-up strategy compared to immediate SLB (4% vs. 50%) [65].

### 1.7. TBC—Procedure Technique

Although TBC for ILD has been used for over a decade, the outcomes have been quite variable. This appears at least partially attributable to the patient heterogeneity and the variability of peri- and intra-procedural factors that showcase the lack of procedural standardization. A systematic review identified differences in practices regarding the number of samples obtained, size of the cryoprobe, routine administration of an agent to prevent bleeding (i.e., topical epinephrine, topical tranexamic acid, others), modality to secure the airway, use of routine post-procedure imaging, and others [66]. General procedural guidelines are now available and provide a framework for standardization to assure good and reproducible outcomes [59,67,68].

The contraindications for TBC include a rapid clinical decline suggestive of an exacerbation of ILD, pulmonary hypertension, bleeding diathesis, and compromised pulmonary function (force vital capacity < 50% of predicted, forced expiratory volume in 1 s <0.80 L or <50% of predicted, diffusing capacity for carbon monoxide < 35% of predicted) [69]. Although the procedure can be performed by experienced users using moderate sedation [70,71], using general anesthesia and a large-size endotracheal tube (≥8.5) or a rigid bronchoscope is recommended. This allows for rapid re-entry into the airway after sampling, decreases the risk of inadvertent adherence of the probe to the airway or the vocal cords, allows for selective contralateral ventilation which may be needed should bleeding occur, and decreases the likelihood of dislodgement of a bronchial blocker with cough [72,73]. 

The samples should be obtained unilaterally from at least two sites as this increases the histopathological yield. This can be from different ipsilateral lobes or from different segments in the same lobe [74]. Careful review of HRCT before the procedure is important to predetermine the expected sampling sites.

There are multiple sizes of cryoprobes available. Reusable cryoprobes come in 2.4 mm and 1.9 mm sizes; disposable cryoprobes come in 2.4 mm, 1.7 mm, and 1.1 mm sizes. Using the 1.9 mm cryoprobe is recommended as it has a similar yield and lower pneumothorax rate compared to the large-size probes [67,69]. Small-size cryoprobes also offer increased maneuverability which may facilitate reaching specific segments. Disposable cryoprobes have not been directly compared.

The bronchoscope is advanced into the lobe and segment of interest and the cryoprobe is then advanced using fluoroscopy until the pleural edge resistance is felt. Using fluoroscopy during TBC is recommended to guide the proximity of the tip of the cryoprobe to the pleura as it is associated with a lower rate of pneumothorax [75]. Once resistance is felt, the cryoprobe is retracted to 1 cm from the pleura where the sampling will occur. This location is more likely to provide adequate sampling, while decreasing the risk of pneumothorax, if the sampling is too peripheral, and the risk of bleeding, if the sampling is too central. The reported cryoprobe freeze time is variable in the literature, with 3–6 s being generally recommended [69]; this may be adjusted based on the visual appearance of the sample obtained. After activation, the bronchoscope, cryoprobe, and sample are removed en bloc. Before any sampling is performed, a prophylactic bronchial blocker is inserted into the ipsilateral lung. This balloon is then inflated immediately upon removal of the bronchoscope and sample to preemptively tamponade the target airways while the sample is processed. The balloon is then deflated under direct bronchoscopic visualization to assure hemostasis, and the procedure is repeated to obtain a total of 4–7 samples [10].

Post procedure, the patient should be monitored for at least three hours. A chest X-ray should be ordered at two hours. A chest X-ray and/or a chest ultrasound should be used immediately if the patient develops symptoms suggestive of a pneumothorax [68].

### 1.8. TBC—Complications

The reported pneumothorax rate in the literature is variable, ranging from 0 to 25%. A recent metanalysis showed a pneumothorax rate of 5%, with 62% requiring chest tube drainage [66]. Bleeding of any severity may occur in up to 30% of cases [5]. Moderate to severe bleeding occurs in 12% of cases and severe bleeding occurs in 0–6% of cases [66,67]. The variability of the bleeding rates seen in the literature is likely due to the lack of consistency in the definitions. To address this issue, the use of a standardized classification for reporting bleeding during bronchoscopy is recommended [76]. Mitigating the occurrence and impact of bleeding can be achieved by careful patient selection and assuring certain procedural factors, such as using a large-size endotracheal tube or rigid bronchoscope, using the 1.9 mm cryoprobe, and using a bronchial blocker. A recent meta-analysis showed an acute exacerbation rate of 1.4% and a 30-day mortality rate of 0.6% after TBC [77]. 

### 1.9. TBC—Competency

As showcased, TBC is a complex procedure that requires careful patient selection, procedural planning, and the ability to manage potential complications to ensure good outcomes. One study reported a high complication rate following the first 25 TBC procedures at a high-volume academic medical center [78]. Expertise in performing TBLB, managing intrabronchial bleeding, and performing endotracheal intubation are the essential requirements before considering performing TBC [73]. Although we cannot ascertain a specific number of procedures or the type of training required to achieve competency, formal training in TBC is recommended to perform the procedure in a standardized, safe, and effective way [59]. Moreover, not only the bronchoscopist, but also the team and the facility must be trained and equipped adequately before offering this procedure. 

## 2. Conclusions

The diagnosis of ILD remains multidisciplinary. Clinical and historical findings coupled with HRCT and serological data provide the necessary information for a confident diagnosis in most cases. Minimally invasive techniques performed via bronchoscopy have varying degrees of utility based on the disease process. BAL is useful in identifying certain specific diseases and in ruling out infection. EBB has a limited role in granulomatous disease. EBUS-TBNA is useful in sarcoidosis, tuberculosis, and malignancy when these affect the mediastinal/hilar structures. TBLB is helpful when there is centrilobular involvement and can aid in the diagnosis of hypersensitivity pneumonitis, organizing pneumonia, and other granulomatous diseases like sarcoidosis. SLB remains the gold standard for histopathological diagnosis in unspecified ILD; however, it is reserved for good surgical candidates. TBC is now considered as a recommended alternative to SLB due to its comparable histopathological yield and favorable safety profile. This procedure should be performed in centers of excellence, by bronchoscopists that have completed adequate training to ensure good outcomes. Continued research into the overall utility of bronchoscopy for the diagnosis of ILD is warranted.

## Figures and Tables

**Table 1:** Cellular patterns of BAL fluids in common DPLDs.

Cellular Patterns of BAL Fluid Analysis	
Lymphocytic Pattern	Hypersensitivity pneumonitis (60–80%) (low CD4:CD8) Early sarcoidosis (40–60%) (elevated CD4:CD8) Idiopathic pulmonary fibrosis (15–30%) Berylliosis (elevated CD4:CD8) Lymphoproliferative disorders Early in pulmonary Langerhans cell histiocytosis
Eosinophilic Pattern	Chronic eosinophilic pneumonia (≥40%) Eosinophilic granulomatosis with polyangitis Acute eosinophilic pneumonia (≥25%) Tropical pulmonary eosinophilia (40–70%) Fungal/endemic pneumonias Idiopathic pulmonary fibrosis (<10%) Sarcoidosis (<25%)
Neutrophilic Pattern	Acute respiratory distress syndrome Bacterial pneumonia Aspiration pneumonitis Idiopathic pulmonary fibrosis (15–40%) Inorganic dust diseases (Asbestosis, silicosis, etc.) Cigarette smoking (<10%) Acute hypersensitivity pneumonitis Advanced sarcoidosis
Mixed Cellular Pattern	Idiopathic pulmonary fibrosis Nonspecific interstitial pneumonia Cryptogenic organizing pneumonia Connective tissue diseases Drug-induced pulmonary diseases

**Table 2:** BAL findings highly suggestive of specific diseases.

Finding in BAL Fluid	Associated Conditions
Malignant cells	Lung cancer, lymphangitic carcinomatosis, pulmonary lymphoma
Fat globules in macrophages Multinucleated giant cells	Lipoid pneumonia
Ferruginous bodies	Asbestosis
Dust particles seen by polarized microscopy	Silicosis
Positive lymphocyte transformation test to beryllium salts	Berylliosis
Hemosiderin-laden macrophages Sequential lavages progressively bloodier	Diffuse alveolar hemorrhage
Eosinophils ≥ 40%	Chronic eosinophilic pneumonia
Eosinophils ≥ 25%	Acute eosinophilic pneumonia
Lipoproteinaceous material (periodic acid–Schiff stain)	Pulmonary alveolar proteinosis
CD1 positive Langerhans cells > 5%/Birbeck granules on electron microscopy	Pulmonary Langerhans cell histiocytosis

## Data Availability

There are no internal data supporting this review article.

## Abbreviations

BAL	Bronchoalveolar lavage
EBB	Endobronchial biopsy
EBUS-TBNA	Endobronchial-ultrasound transbronchial needle aspiration
ILD	Interstitial lung disease
DPLD	Diffuse parenchymal lung disease
MDD	Multidisciplinary meeting
SLB	Surgical lung biopsy
TBLB	Transbronchial lung biopsy
TBC	Transbronchial lung cryobiopsy
